# A Comparative Analysis of Liver Injury Markers in Post-COVID Syndrome among Elderly Patients: A Prospective Study

**DOI:** 10.3390/jcm13041149

**Published:** 2024-02-18

**Authors:** Adrian Vasile Bota, Felix Bratosin, Satya Sai Sri Bandi, Iulia Bogdan, David Vladut Razvan, Ana-Olivia Toma, Mirela Florica Indries, Andrei Nicolae Csep, Coralia Cotoraci, Mihaela Prodan, Felicia Marc, Flavia Ignuta, Iosif Marincu

**Affiliations:** 1 Methodological and Infectious Diseases Research Center, Department of Infectious Diseases, “Victor Babes” University of Medicine and Pharmacy Timisoara, Eftimie Murgu Square 2, 300041 Timisoara, Romania; bota.adrian@student.uvvg.ro (A.V.B.); felix.bratosin@umft.ro (F.B.); iulia-georgiana.bogdan@umft.ro (I.B.); vladut-razvan.david@umft.ro (D.V.R.); flavia.ignuta@umft.ro (F.I.); imarincu@umft.ro (I.M.); 2 Doctoral School, “Victor Babes” University of Medicine and Pharmacy Timisoara, Eftimie Murgu Square 2, 300041 Timisoara, Romania; mihaela.prodan@umft.ro; 3 Department of Hematology, Faculty of Medicine, “Vasile Goldis” Western University, Bulevardul Revolutiei 94, 310025 Arad, Romania; rector_vg@uvvg.ro; 4 Discipline of Infectious Diseases, “Victor Babes” University of Medicine and Pharmacy Timisoara, Eftimie Murgu Square 2, 300041 Timisoara, Romania; 5 Malla Reddy Institute of Medical Sciences, Suraram Main Road 138, Hyderabad 500055, India; sssbandi09@gmail.com; 6 Discipline of Dermatology, “Victor Babes” University of Medicine and Pharmacy Timisoara, Eftimie Murgu Square 2, 300041 Timisoara, Romania; 7 Department of Psycho-Neuroscience and Recovery, Faculty of Medicine and Pharmacy, University of Oradea, Strada Universitatii 1, 410087 Oradea, Romania; mirela.indries@gmail.com; 8 Department of Plastic Surgery, “Victor Babes” University of Medicine and Pharmacy Timisoara, Eftimie Murgu Square 2, 300041 Timisoara, Romania; 9 Department of Medical Sciences, Faculty of Medicine and Pharmacy, University of Oradea, 410073 Oradea, Romania

**Keywords:** SARS-CoV-2, COVID-19, Long COVID

## Abstract

Background: In the wake of the global COVID-19 pandemic, understanding its prolonged impact on vulnerable populations has become a critical area of investigation. This study aimed to elucidate the distinctive post-acute sequelae of SARS-CoV-2 infection (PASC) and liver injury in Romania’s elderly population, hypothesizing unique demographic, clinical, and healthcare factors influencing the manifestation. Methods: A longitudinal design was employed, enrolling COVID-19 patients from the Victor Babes Hospital for Infectious Diseases and Pulmonology in Timisoara, Romania. Participants were stratified into three groups based on age and Long COVID status. The study focused on a variety of demographic, clinical, and biological parameters, including liver function tests, to assess the trajectory and severity of liver injury over six months post discharge. Results: Involving 238 participants, the study revealed a significant increase in the duration of hospitalization for those over 65 (15.8 ± 8.2 days) compared to younger groups (*p* < 0.001). Notably, elderly Long COVID patients exhibited a marked elevation in liver enzymes post discharge, with median ΔALT and ΔAST of 24.1 U/L and 30.2 U/L, respectively, suggesting ongoing liver injury (*p* < 0.001). Significant metabolic disruptions were observed, with the ΔFasting glucose showing a substantial median decrease of 21.1 mmol/L in the elderly group (*p* < 0.001). A pronounced reduction in ΔGGT (16.7 U/L) and ΔLDH (48.7 U/L) was noted, indicating a recovery in liver function and reduced tissue damage (*p* < 0.001). Coagulation profiles and liver fibrosis risk scores, particularly ΔFIB-4 and ΔAPRI, also significantly improved post discharge, indicating a reduced risk of ongoing liver complications. Conclusion: This study confirms the hypothesis of more severe PASC and liver injury among the elderly Romanian population. Significant improvements post discharge suggest a degree of recovery, yet the persistent alterations in liver enzymes, glucose metabolism, and fibrosis risk scores call for continued monitoring and tailored management strategies.

## 1. Introduction

The COVID-19 pandemic, caused by the coronavirus SARS-CoV-2, has had a profound impact on certain populations such as those with cancer, the immunocompromised, and the elderly [1,2,3,4,5]. The elderly population has been particularly vulnerable to severe outcomes of the infection. A systematic review and meta-analysis found that COVID-19 can lead to a wide range of long-term effects, affecting up to 80% of patients post infection, with symptoms including fatigue, headache, and dyspnea [6].

Long COVID, also known as post-acute sequelae of SARS-CoV-2 infection (PASC), is characterized by persistent symptoms and complications following the initial recovery from COVID-19, reported in various populations with or without comorbid conditions [7,8,9,10,11,12]. Lenz et al. highlighted that more than 50 long-term symptoms of COVID-19 have been identified, affecting various systems including cardiac, pulmonary, and neurologic systems, with elderly individuals and oncologic patients at increased risk due to factors like older age and the presence of comorbidities [13,14,15,16].

The manifestations of Long COVID in the elderly are distinct, with studies demonstrating that older adults are more likely to experience symptoms like fatigue and dyspnea, along with pulmonary and liver impairment [17,18,19]. Additionally, post-infection cognitive impairments are a significant concern, as evidenced by a study that found an increased risk of cognitive decline, especially in severe COVID-19 cases among the elderly or those with pre-existing psychiatric conditions [20,21,22,23].

Despite the global nature of the pandemic, there is a continuous need for focused research on the long-term effects of COVID-19 in the elderly, particularly in specific geographic contexts like Romania, a country that was significantly affected by the pandemic, and with a case–fatality rate of 3.1% [24,25,26,27]. This lack of region-specific data highlights a critical gap in understanding the unique challenges and needs of the elderly in different cultural and healthcare settings or in the context of low COVID-19 vaccination acceptance, with a complete scheme of 43% as of mid 2023 [28,29].

This study hypothesizes that the post-acute sequelae of SARS-CoV-2 infection manifest distinctly in the elderly population in Romania, influenced by unique demographic, clinical, and healthcare factors. Moreover, it is hypothesized that liver injury is more prevalent among the elderly patients, with varying degrees of severity. The primary objective is to conduct a comprehensive analysis of the clinical and laboratory characteristics and severity of PASC symptoms in this demographic over a two-year period. Additionally, the study aims to identify demographic and clinical predictors of long-term sequelae.

## 2. Materials and Methods

### 2.1. Research Design and Ethical Considerations

The study employed a longitudinal research design to explore the liver function tests among COVID-19 patients from the onset of their diagnosis and subsequently over a defined period of six months post discharge. This approach was taken to understand the trajectory of these parameters, especially in the context of post-COVID syndrome. Patients were recruited after admission to the Victor Babes Hospital for Infectious Diseases and Pulmonology in Timisoara, Romania, affiliated with the Victor Babes University of Medicine and Pharmacy from Timisoara. Adhering to the strictest ethical standards, the research was sanctioned by the Local Commission of Ethics for Scientific Research, which is in alignment with the EU GCP Directives 2005/28/EC, ICH guidelines, and the principles specified in the Declaration of Helsinki.

### 2.2. Inclusion Criteria and Definitions

Eligible patients were adults aged 18 and above, who were admitted to hospitals for SARS-CoV-2 infection, who gave their consent for study participation. For each month of the study, a specific number of patients were targeted to ensure a substantial sample size for the entire research duration. Patients vaccinated for COVID-19 were excluded from the study to avoid the potential modifying effect of the vaccine. Patients with a history of chronic lung disease and those with cancer were also excluded. Another exclusion criterium was set for patients who took medications that influence the PT. Patients were matched by COVID-19 severity, age, and gender.

Individuals with chronic liver diseases, including hepatitis B and C, were excluded from participation, in order to eliminate any confounding variables that could obscure the specific impact of COVID-19 on liver function. To uphold the integrity of this exclusion criteria, testing for hepatitis B and C was conducted on all potential participants prior to their inclusion in the study. Abdominal ultrasound was among the routine investigations performed in these patients

Post-acute COVID-19 syndrome refers to a collection of symptoms that persist beyond four weeks from the onset of the initial acute symptoms of the SARS-CoV-2 virus. Despite biochemical evidence that viral replication ceases about four weeks after initial infection, some individuals continue to experience lingering symptoms. The Center for Disease Control (CDC) has formulated the term “post-COVID syndrome” to encompass health issues like Long COVID and persistent post-COVID syndrome (PPCS), multiorgan effects of COVID-19, and the impacts of COVID-19 treatment or hospitalization [30]. Common manifestations of this syndrome include fatigue, brain fog, dyspnea, autonomic dysfunction, and various symptoms related to cardiovascular, pulmonary, renal, neuropsychiatric, endocrine, and hematologic systems. The duration, severity, and specific manifestations can vary widely among patients.

### 2.3. Variables

In the current study, various demographic, clinical, and biological parameters were analyzed. The baseline characteristics examined included age, with mean and standard deviation noted; Body Mass Index (BMI) as mean ± standard deviation; gender, specifically the percentage of men in each group; smoking status; frequency of alcohol use; and the prevalence of comorbidities such as cardiovascular disease, metabolic disease, autoimmune disease, chronic kidney disease, and other conditions. The Charlson Comorbidity Index (CCI) score was recorded, noting the number of participants with a score of ≥2. Details of oxygen supplementation, COVID-19 severity categorized as mild, moderate, and severe, days of hospitalization, and Intensive Care Unit (ICU) admission were also included.

The study also thoroughly assessed biological parameters during hospitalization. These included Fasting Glucose (mmol/L), Alanine Aminotransferase (ALT, U/L), Aspartate Aminotransferase (AST, U/L), Alkaline Phosphatase (ALP, U/L), Serum Albumin (g/dL), Total Proteins (g/dL), total bilirubin (g/dL), Gamma-Glutamyl Transpeptidase (GGT, U/L), Lactate Dehydrogenase (LDH, U/L), Prothrombin Time (PT, seconds), Activated Partial Thromboplastin Time (APTT, seconds), Fibrosis-4 (FIB-4) Score, Nonalcoholic Fatty Liver Disease Fibrosis Score (NFS), and Aspartate Aminotransferase to Platelet Ratio Index (APRI). These variables were compared across three distinct groups: Long COVID patients under 65 years, Long COVID patients 65 years and older, and a control group without Long COVID. 

### 2.4. Statistical Analysis

Data management and analysis were conducted utilizing the statistical software SPSS version 26.0 (SPSS Inc., Chicago, IL, USA). The sample size was calculated based on a convenience sampling method, with a minimum of 180 patients at a 95% confidence level and 10% margin of error. Continuous variables with a Gaussian distribution were represented as mean ± standard deviation (SD), while non-Gaussian data were reported as median (IQR). Categorical variables were expressed in terms of frequencies and percentages. To analyze the changes between more than two means of continuous variables with a normal distribution, the ANOVA test was utilized, while for those with a non-normal distribution, the Kruskal–Wallis test was employed. The Chi-square test was utilized for the categorical variables. A regression analysis evaluated the liver injury risk. A *p*-value threshold of less than 0.05 was set for statistical significance. All results were double-checked to ensure accuracy and reliability.

## 3. Results

The Body Mass Index showed no significant differences among the groups, with mean values of 24.2, 24.8, and 23.9 for the <65 years, ≥65 years, and No Long COVID groups, respectively (*p* = 0.618). Gender distribution, represented by the percentage of men in each group, did not show a significant difference across the groups (*p* = 0.899). Similarly, lifestyle factors such as smoking and frequent alcohol use did not differ significantly among the groups, with *p*-values of 0.171 and 0.307, respectively.

When analyzing comorbidities, no significant differences were found among the groups for cardiovascular disease (*p* = 0.540), metabolic disease (*p* = 0.251), autoimmune disease (*p* = 0.637), chronic kidney disease (*p* = 0.239), and other comorbidities (*p* = 0.287). A Charlson Comorbidity Index (CCI) score of ≥2 also showed no significant difference (*p* = 0.222), indicating a similar comorbidity burden across the groups. Oxygen supplementation, a crucial clinical intervention for COVID-19 patients, did not significantly differ among the groups (*p* = 0.055), indicating similar respiratory support needs. The severity of COVID-19, categorized as mild, moderate, or severe, also did not show a significant difference across the groups (*p* = 0.506).

However, the days of hospitalization exhibited a significant difference, with the Long COVID ≥65 years group having a longer duration of hospital stay (15.8 days) compared to the <65 years (11.3 days) and No Long COVID groups (13.2 days), with a *p*-value of <0.001, suggesting that older patients with Long COVID had a more prolonged hospitalization period. Intensive Care Unit (ICU) admission rates among the groups did not show a significant difference (*p* = 0.221), with a total of 19.7% in the elderly group, down to 10.0% in the No Long COVID group, as presented in Table 1.

Fasting glucose levels were notably higher in both Long COVID groups compared to the control group, with median values of 132.4 mmol/L and 147.8 mmol/L for the <65 and ≥65 groups, respectively, versus 112.5 mmol/L in the No Long COVID group, indicating a statistically significant difference (*p* < 0.001). Liver enzymes, including Alanine Aminotransferase (ALT) and Aspartate Aminotransferase (AST), were elevated in Long COVID patients, with the ≥65 years group showing the highest levels. The median ALT and AST levels in the ≥65 group were 82.3 U/L and 92.5 U/L, respectively, compared to the control group’s 47.4 U/L and 52.3 U/L (*p* = 0.006 and *p* < 0.001, respectively).

Alkaline Phosphatase (ALP) and Gamma Glutamyl Transpeptidase (GGT), other markers for liver health, were similarly elevated in the Long COVID groups, particularly in those aged 65 and above, suggesting possible cholestasis or biliary injury (*p* < 0.001 for both). Total bilirubin levels were also significantly higher in the Long COVID groups, particularly in the older patients, indicating possible liver dysfunction (*p* = 0.002).

Interestingly, Serum Albumin and Total Proteins were lower in the Long COVID groups, especially in the elderly, reflecting potential nutritional deficiencies or liver synthetic dysfunction (*p* = 0.044 and *p* = 0.020, respectively). The coagulation markers, Prothrombin Time (PT) and Activated Partial Thromboplastin Time (APTT), were prolonged in Long COVID patients, especially those aged 65 and above, indicating a disturbed coagulation profile possibly related to liver dysfunction or systemic inflammation (*p* = 0.039 and *p* = 0.010, respectively). Furthermore, the study utilized scores like FIB-4, NFS, and APRI to assess liver fibrosis risk. All three scores were significantly higher in patients with Long COVID, particularly in the elderly group, suggesting an increased risk of liver fibrosis associated with Long COVID (*p* < 0.001 for FIB-4 and NFS, *p* < 0.001 for APRI), as seen in Table 2.

Fasting glucose levels remained elevated six months after hospitalization in both Long COVID groups compared to the controls, with median levels significantly higher in the ≥65 group (126.7 mmol/L) than the <65 group (119.2 mmol/L) and the No Long COVID group (97.8 mmol/L), indicating a persistent disruption in glucose metabolism, especially in the elderly (*p* < 0.001). Liver enzymes such as Alanine Aminotransferase (ALT) and Aspartate Aminotransferase (AST) continued to show elevated levels in the Long COVID groups. The ≥65 group exhibited the highest median ALT and AST levels, 58.2 U/L and 62.3 U/L, respectively, compared to the control group’s 28.5 U/L and 33.9 U/L, suggesting ongoing liver stress or injury (*p* < 0.001 for both).

Alkaline Phosphatase (ALP) levels were significantly higher in Long COVID patients, especially among those aged 65 and above, with a median of 164.3 U/L compared to 110.2 U/L in the control group, which may indicate persistent biliary or liver cell damage (*p* < 0.001). Similarly, Gamma Glutamyl Transpeptidase (GGT) levels were elevated, particularly in the older Long COVID group, suggesting continued cholestatic or hepatocellular injury (*p* < 0.001).

Interestingly, while the Serum Albumin and Total Protein levels were closer to the normal range, they remained significantly different across the groups, with the elderly Long COVID group showing lower median values compared to the control group, potentially indicating ongoing nutritional or synthetic liver issues (*p* = 0.038 and *p* = 0.022, respectively). Coagulation profiles, as indicated by Prothrombin Time (PT) and Activated Partial Thromboplastin Time (APTT), remained altered in Long COVID patients, especially in those aged 65 and above, suggesting a persistent pro-thrombotic state or ongoing liver dysfunction affecting coagulation (*p* < 0.001 for both).

Fibrosis risk scores like FIB-4 and APRI remained higher in the Long COVID groups, particularly in the elderly, indicating a sustained risk of liver fibrosis six months post infection (*p* = 0.010 for FIB-4; *p* = 0.055 for APRI). However, the NFS score did not show a statistically significant difference between the groups, suggesting a more nuanced impact on liver fibrosis risk (*p* = 0.073), as presented in Table 3.

For patients under 65 years, significant improvements were observed in several biochemical markers over six months. Fasting glucose levels decreased from a median of 132.4 mmol/L at admission to 119.2 mmol/L after six months, indicating a significant reduction in glucose levels post recovery (*p* < 0.001). Liver enzymes such as Alanine Aminotransferase (ALT) and Aspartate Aminotransferase (AST) also showed significant decreases, suggesting a recovery in liver function (*p* < 0.001 for ALT and AST). Alkaline Phosphatase (ALP) levels showed a modest but significant reduction (*p* = 0.004), while Gamma Glutamyl Transpeptidase (GGT) and Lactate Dehydrogenase (LDH) levels also decreased significantly (*p* = 0.001 and *p* = 0.042, respectively), indicating an overall improvement in liver health and enzyme activity.

Similarly, in the elderly group (≥65 years), significant decreases were observed in fasting glucose, ALT, AST, ALP, GGT, and LDH levels (all *p* < 0.001), suggesting a general improvement in metabolic health and liver function. Notably, Serum Albumin levels increased significantly in both age groups, indicating an improvement in protein synthesis and nutritional status (*p* < 0.001 for both groups). Total bilirubin levels also decreased significantly in the older group (*p* = 0.033), further confirming the trend towards recovery.

Coagulation profiles, assessed through Prothrombin Time (PT) and Activated Partial Thromboplastin Time (APTT), showed significant improvements in the younger group (*p* = 0.010 for APTT) and a trend towards improvement in the older group, indicating a recovery from the pro-thrombotic state observed during acute infection. Interestingly, the study also observed significant reductions in liver fibrosis risk scores such as FIB-4 and NFS in both age groups (*p* < 0.001 for FIB-4 in both groups; *p* < 0.001 for NFS in the younger group; and *p* = 0.010 in the older group), suggesting a decrease in the risk of developing liver fibrosis post COVID-19. The AST to Platelet Ratio Index (APRI) also showed significant improvement, especially in the younger group (*p* = 0.003), further supporting the trend of liver recovery, as presented in Table 4.

A significant decrease in fasting glucose levels was observed across all groups, with the most substantial change noted in the ≥65 years group (median decrease of 21.1 mmol/L), compared to the <65 years group (13.2 mmol/L) and the control group (14.7 mmol/L), *p* < 0.001. This suggests an overall improvement in metabolic control post infection, which was especially prominent in the elderly. The liver enzymes ALT and AST also showed significant decreases, indicative of liver function recovery. The ≥65 years group experienced a more pronounced decrease in ALT (24.1 U/L) compared to the <65 years group (12.0 U/L) and the control group (18.9 U/L), *p* < 0.001. Similarly, AST decreased more in the ≥65 years group (30.2 U/L) compared to the <65 years group (19.4 U/L), both significantly more than the control group (18.4 U/L), *p* < 0.001.

The Alkaline Phosphatase (ALP) and Gamma Glutamyl Transpeptidase (GGT) changes also reflected significant liver function recovery post discharge, particularly in the elderly and Long COVID groups, with changes of 30.4 U/L and 16.7 U/L, respectively, *p* < 0.001 for both. The Lactate Dehydrogenase (LDH) levels showed substantial decreases, especially in the Long COVID groups, indicating a general decrease in tissue damage or inflammation. Interestingly, while the total bilirubin change was significant (*p* = 0.034), it was the least among the groups, suggesting a more nuanced recovery pattern for bilirubin metabolism.

**Figure 1:**
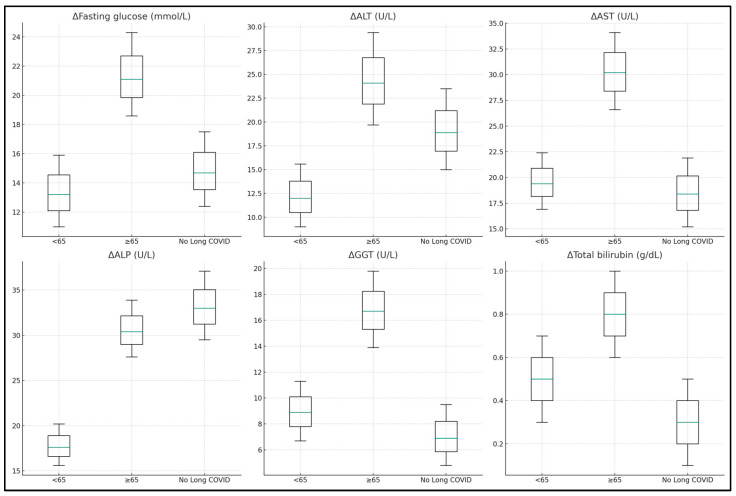
Laboratory value differences at admission and six months post discharge.

**Figure 2:**
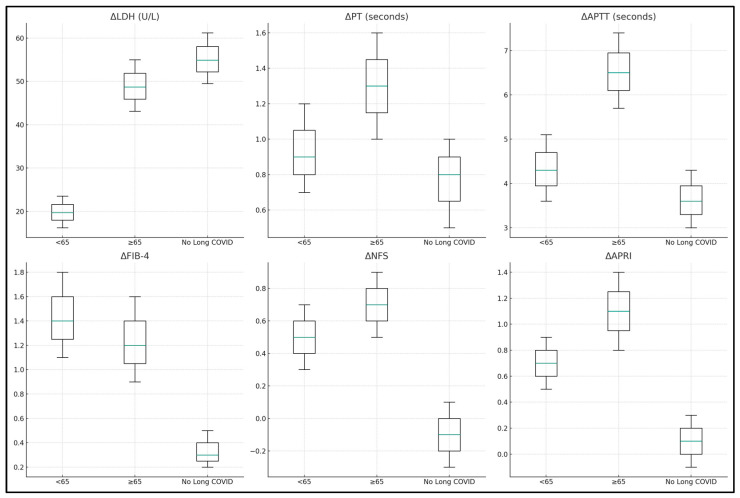
Laboratory value differences at admission and six months post discharge.

Coagulation profiles, measured through changes in Prothrombin Time (PT) and Activated Partial Thromboplastin Time (APTT), showed significant improvements across all groups, with the elderly group showing a higher median change, indicating a move towards normal coagulation function post COVID. Fibrosis risk scores, FIB-4 and NFS, showed notable decreases, particularly in the Long COVID groups, suggesting a reduced risk of liver fibrosis development post COVID. The AST to Platelet Ratio Index (APRI) also decreased significantly, especially in the elderly group (1.1), indicating an overall improvement in liver health and reduced fibrosis risk, as presented in Table 5 and Figure 1 and Figure 2.

Age showed a significant positive association with liver injury severity, with each additional year increasing the severity score by 0.048 (*p* < 0.001). Gender did not significantly predict liver injury severity, with a coefficient of −0.107 for males compared to females (*p* = 0.558). Body Mass Index (BMI) also did not show a significant relationship with liver injury severity (*p* = 0.148). In terms of clinical factors, cardiovascular disease was associated with an increased severity of liver injury, with a coefficient of 0.429 (*p* = 0.046), while chronic kidney disease showed a similar trend but did not reach statistical significance (*p* = 0.069). Smoking status and a Charlson Comorbidity Index (CCI) score of ≥2 were not significant predictors.

Several biological parameters changed significantly in association with liver injury severity. Increases in AST and ALP were associated with increased liver injury severity, with coefficients of 0.036 (*p* < 0.001) and 0.049 (*p* < 0.001), respectively. Total bilirubin showed a substantial positive association with a coefficient of 1.198 (*p* = 0.003), indicating that higher changes in bilirubin levels were associated with greater liver injury severity. Conversely, higher changes in Serum Albumin levels were associated with a decrease in liver injury severity, with a coefficient of −1.503 (*p* = 0.005). Changes in GGT, LDH, PT, APTT, NFS, and APRI also significantly predicted liver injury severity, with varying coefficients. 

**Figure 3:**
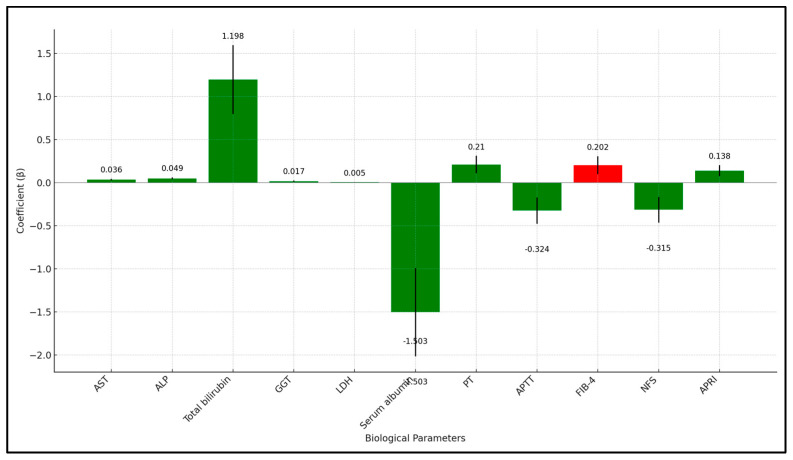
Regression analysis results (laboratory values).

Treatment factors were also explored. Patients who received oxygen supplementation did not have a significant change in liver injury severity (*p* = 0.489), while increased enzyme activities were demonstrated in patients hospitalized in the ICU, with a coefficient of 0.483 (*p* = 0.042), as presented in Table 6 and Figure 3.

## 4. Discussion

### 4.1. Important Findings and Literature Review

The study’s results highlight the complex interplay between demographic characteristics, clinical factors, and biological markers in the context of Long COVID. Despite no significant differences in BMI, gender, and lifestyle factors such as smoking and alcohol use, the marked elevation in fasting glucose levels and liver enzymes like ALT and AST in Long COVID patients, particularly in those aged 65 and above, points to a persistent metabolic disruption and liver stress. This finding is alarming as it suggests a prolonged impact of the virus, necessitating ongoing monitoring and intervention strategies tailored to metabolic and liver health. The elevated levels of ALP, GGT, and total bilirubin further indicate a spectrum of liver injuries, from cholestasis to liver cell damage, which could have long-term implications on patient health.

The study’s use of coagulation markers and liver fibrosis scores provides a comprehensive overview of liver health post infection. The prolonged PT and APTT times, coupled with the increased scores for FIB-4, NFS, and APRI, highlight an altered coagulation profile and an increased risk of liver fibrosis in Long COVID patients. These findings are particularly significant as they indicate not just acute liver injury but also the potential for chronic liver conditions post COVID, raising the need for long-term liver health management in these patients.

The regression analysis further elucidates the predictors of liver injury severity. Age emerges as a critical factor, with each additional year contributing to increased severity. The significant associations of liver injury with cardiovascular disease and the Charlson Comorbidity Index (CCI) score suggest that pre-existing health conditions may exacerbate the liver’s response to COVID-19. In contrast, gender and BMI showed no significant relationship with liver injury severity, suggesting that other factors play a more crucial role in the liver’s response to the virus.

Treatment variables such as oxygen supplementation and ICU admission reveal the complexity of managing COVID-19 patients. While oxygen supplementation did not significantly change liver injury severity, increased enzyme activities were demonstrated in patients hospitalized in the ICU, highlighting the intense impact of severe COVID-19 cases on liver health. These insights are invaluable for healthcare providers as they underscore the importance of comprehensive care that addresses not only the respiratory aspects of COVID-19 but also its systemic impacts, including liver health.

Some studies observed increased ALT, GGT, and ALP levels within 14 days post discharge and two months after initial COVID-19 infection [31], while others noted potential elevation of these liver markers up to three months post recovery [32]. This liver marker alteration, indicative of hepatocyte damage and systemic inflammation, is evidenced by increased levels of various interleukins and IFN-λ in hospitalized patients [33]. Severe COVID-19 cases, similarly to our findings, show raised AST, ALT, and GGT levels with reduced albumin, although mean albumin remained normal in different studies [34]. AST’s role as a liver marker is complex due to its muscle production, especially given that Long COVID-19 patients often face fatigue and muscle weakness [35]. Liver stiffness and viscosity, associated with lung injury during acute COVID-19, were notably higher in patients with exacerbated post-COVID-19 symptoms, linking liver viscosity to inflammation and hepatic steatosis [9,36], as indicated as well by our findings in the liver fibrosis parameters FIB-4, APRI, and NFS.

Despite ferritin being a significant inflammatory marker, all study groups exhibited elevated mean ESRs, hinting at ongoing systemic inflammation [32]. The relationship between ALT and AST in hospitalized COVID-19 patients further supports liver damage’s viral etiology rather than muscle degradation or inflammation [37]. Persistent inflammatory response and ACE2 expression in cholangiocytes, alongside the ACE2-mediated viral invasion, disrupted immune balance, and drug-induced hepatotoxicity, are implicated in the pathogenesis of long-term COVID-19 liver injury [38]. Nevertheless, patients with severe COVID-19 often require multiple drug therapies, many with hepatotoxic potential, exacerbating long-term liver damage risks [39].

Although our study excluded the patients with chronic liver disease to avoid any confounding effects, it is important to underline this important group of comorbid conditions, with potentially worsened COVID-19 outcomes [40]. In a meta-analysis that studied approximately 25 thousand COVID-19 patients, the pooled prevalence of chronic liver disease (CLD) was 3%, indicating that a significant subset of patients might suffer from exacerbated liver impairment due to the viral infection [41]. Particularly vulnerable are those with pre-existing liver conditions such as cirrhosis, alcoholic liver disease (ALD), hepatocellular carcinoma (HCC), and metabolic-associated fatty liver disease (MAFLD), as these conditions increase the risk of severe COVID-19 outcomes [42]. Patients with cirrhosis demonstrated a higher mortality rate, with figures rising from 19% in Child–Pugh class A to 51% in class C, and those with cirrhosis also had increased rates of ICU admission and mechanical ventilation, underscoring the severity of their condition [43].

MAFLD has emerged as a significant concern, with studies showing it as an independent risk factor for severe COVID-19, leading to higher rates of ICU admission and mechanical ventilation [44]. Notably, another study found MAFLD to be an independent predictor for these severe outcomes [45]. In contrast, ALD has been independently linked to increased COVID-19 mortality, with one study reporting it as an independent risk factor for higher 30-day mortality post COVID-19 [46]. These findings highlight the critical influence of liver health on the course and severity of COVID-19, emphasizing the need for careful management of patients with pre-existing liver conditions.

Another area of concern regarding our study findings regards the reliability of the FIB-4 index in individuals older than 65 years, which is underscored by research indicating that aging significantly affects FIB-4 calculations, leading to potential overestimation of liver fibrosis risk in this demographic [47]. This overestimation is due in part to age-related changes in liver enzymes and the inherent age weighting within the FIB-4 formula itself. Studies highlight that the distribution of FIB-4 values shifts with age, with a higher proportion of elderly individuals crossing the threshold for high-risk fibrosis scores, even in the absence of significant liver disease. For instance, a large study examining over 75,000 residents who underwent health checkups found that a significant percentage of individuals aged 70 years and older had FIB-4 scores indicative of a high risk of liver fibrosis, suggesting that the cutoff values for FIB-4 might need reconsideration to avoid unnecessary referrals to hepatologists among the elderly population.

Further examination into the performance of FIB-4 in elderly populations without pre-existing diseases also supports the notion of age affecting FIB-4 index accuracy. An analysis on a cohort of healthy individuals who underwent annual health checkups for ten consecutive years indicated that aging impacts the FIB-4 index, necessitating a critical reevaluation of its use for liver fibrosis screening in older adults [48]. Additionally, the poor performance of FIB-4 in elderly individuals at risk for chronic liver disease has been documented, pointing out the limitations of FIB-4 in accurately reflecting liver fibrosis stage in this age group and the potential for misclassification and overestimation of fibrosis risk [49]. In the context of our study’s findings, which revealed significant liver injury and metabolic disruptions among elderly COVID-19 survivors, these insights suggest that while FIB-4 and other liver fibrosis scores showed improvement post discharge, the interpretation of these scores, particularly in older adults, should be approached with caution.

### 4.2. Study Limitations

This study’s design, centered around assessing liver function post-SARS-CoV-2 infection over six months in a Romanian elderly demographic, inherently encounters limitations. The longitudinal nature and specific focus on an older age group may limit the generalizability of findings to broader populations and other geographical contexts. The exclusion of vaccinated individuals and those with chronic lung diseases or cancer, while necessary to isolate the effects of the virus, further narrows the applicability of results. Additionally, the reliance on convenience sampling and the inherent variability in post-COVID sequelae symptoms present challenges in establishing definitive causal relationships. Our study’s monocentric nature inherently limits the size of available data, precluding a more extensive and diverse sample size that could potentially enhance the findings’ generalizability. Additionally, the absence of a comparison group without COVID-19 in our study design restricts our ability to draw more definitive contrasts in liver function changes between affected and unaffected elderly populations. Among other factors, weight changes were not tracked during the study period, potentially leaving room for unexplored worsening factors among patients with affected liver function. Moreover, the study’s scope, confined to a single center, may not capture the full spectrum of clinical practices and patient experiences across different healthcare settings.

Due to the lack of baseline liver function assessments prior to SARS-CoV-2 infection, our study faces limitations in conclusively attributing liver damage to the virus. This constraint, compounded by our inability to conduct comprehensive laboratory tests without external funding, introduces potential biases in interpreting the impact of Long COVID on liver health. Despite this limitation, we utilized established normal ranges for the laboratory values we measured, allowing us to compare our data against these benchmarks. Lastly, we acknowledge the concern about using FIB-4 and APRI for assessing liver fibrosis, especially since they are not typically applied to acute liver injury. Our study was exploratory in nature, aiming to investigate if these formulas could have relevance in the context of Long COVID (PASC), a syndrome unknown when FIB-4 and APRI were developed. Given PASC’s novel and diverse impacts, including on liver function, we sought to determine if these assessments might offer insights beyond their traditional use, exploring new potential utilities in the face of this complex condition. 

## 5. Conclusions

The study’s findings substantiate the hypothesis that the elderly Romanian population experiences more pronounced post-acute sequelae of SARS-CoV-2 infection and liver injury, indicating a demographic significantly impacted by the long-term effects of COVID-19. The observed persistent elevations in liver enzymes and glucose levels in the elderly post discharge highlight a continued vulnerability and the necessity for ongoing medical surveillance and intervention. Despite the noted improvements in metabolic and liver functions over time, the sustained alterations and risk scores for liver fibrosis signal a critical need for a comprehensive, multidisciplinary approach to post-COVID care in this demographic. These insights underscore the importance of tailored healthcare strategies to mitigate the long-term impacts of COVID-19, emphasizing the necessity for robust healthcare systems ready to address the complex, evolving needs of the aging population affected by the pandemic. Further research is imperative to unravel the underlying mechanisms, optimize treatment protocols, and improve the quality of life for those suffering from prolonged COVID-19 consequences. 

## Figures and Tables

**Table 1:** Comparison of baseline characteristics between cases and controls.

Variables	Long COVID <65 Years (*n* = 117)	Long COVID ≥65 Years (*n* = 71)	No Long COVID (n = 50)	*p*-Value
Background data				
BMI, kg/m^2^ (mean ± SD)	24.2 ± 4.9	24.8 ± 5.7	23.9 ± 5.5	0.618
Gender (men)	65 (55.6%)	37 (52.1%)	27 (54.0%)	0.899
Smoking	45 (38.5%)	20 (28.2%)	22 (44.0%)	0.171
Frequent alcohol use	8 (6.8%)	8 (11.3%)	7 (14.0%)	0.307
Comorbidities				
Cardiovascular disease	43 (36.8%)	31 (43.7%)	22 (44.0%)	0.540
Metabolic disease	18 (15.4%)	12 (16.9%)	13 (26.0%)	0.251
Autoimmune disease	4 (3.4%)	2 (2.8%)	3 (6.0%)	0.637
Chronic kidney disease	5 (4.3%)	7 (9.9%)	5 (10.0%)	0.239
Others	6 (5.1%)	5 (7.0%)	6 (12.0%)	0.287
CCI score (≥2)	26 (22.2%)	25 (35.2%)	16 (32.0%)	0.222
Oxygen supplementation				0.055
No supplementation	11 (9.4%)	3 (4.2%)	7 (14.0%)	
Non-invasive ventilation	88 (75.2%)	46 (64.8%)	33 (66.0%)	
Invasive ventilation	18 (15.4%)	22 (31.0%)	10 (20.0%)	
COVID-19 severity				0.506
Mild	40 (34.2%)	18 (25.4%)	15 (30.0%)	
Moderate	43 (36.8%)	25 (35.2%)	21 (42.0%)	
Severe	34 (29.1%)	28 (39.4%)	14 (28.0%)	
Disease outcomes				
Days of hospitalization (mean ± SD)	11.3 ± 6.9	15.8 ± 8.2	13.2 ± 8.0	<0.001
ICU admission	14 (12.0%)	14 (19.7%)	12 (10.0%)	0.221

SD—Standard Deviation; BMI—Body Mass Index; ICU—Intensive Care Unit; CCI—Charlson Comorbidity Index.

**Table 2:** Comparison of biological parameters during hospitalization between cases and controls.

Variables (Median IQR)	Normal Range	Long COVID <65 Years (*n* = 117)	Long COVID ≥65 Years (*n* = 71)	No Long COVID (n = 50)	*p*-Value
Fasting glucose (mmol/L)	60–125	132.4 (121.3–143.7)	147.8 (136.5–158.2)	112.5 (101.9–123.4)	<0.001
ALT (U/L)	7–35	61.7 (51.2–72.9)	82.3 (71.8–93.1)	47.4 (37.6–56.5)	0.006
AST (U/L)	10–40	71.2 (61.4–81.9)	92.5 (82.7–103.6)	52.3 (42.1–61.8)	<0.001
ALP (U/L)	40–130	163.5 (152.3–175.8)	194.7 (182.4–207.9)	143.2 (131.7–154.3)	<0.001
Serum albumin (g/dL)	3.4–5.4	2.6 (2.4–2.9)	2.1 (1.9–2.4)	3.2 (2.9–3.5)	0.044
Total proteins (g/dL)	6.0–8.3	6.7 (6.3–7.2)	5.9 (5.4–6.5)	7.3 (6.8–7.9)	0.020
Total bilirubin (g/dL)	0.3–1.2	1.8 (1.5–2.2)	2.4 (2.0–2.9)	1.1 (0.9–1.3)	0.002
GGT (U/L)	0–30	45.3 (39.8–52.1)	58.6 (51.9–66.4)	31.5 (25.7–38.2)	<0.001
LDH (U/L)	140–280	312.4 (295.1–331.7)	364.5 (345.9–384.8)	265.3 (248.6–283.7)	<0.001
PT (seconds)	11.0–13.5	15.2 (14.6–15.9)	16.7 (16.0–17.5)	13.4 (12.8–14.1)	0.039
APTT (seconds)	30–40	43.8 (41.5–46.4)	49.3 (46.9–52.1)	36.7 (34.3–39.2)	0.010
FIB-4	1.45–3.25	3.3 (2.0–4.7)	3.9 (2.5–4.4)	1.7 (1.4–2.0)	<0.001
NFS	≤1.5	−0.4 (−0.7–−0.1)	0.2 (-0.1–0.6)	−1.6 (−1.9–−1.3)	<0.001
APRI	0.5–1.5	1.8 (1.0–2.5)	2.6 (1.3–3.9)	0.8 (0.6–1.0)	<0.001

IQR—Interquartile Range; ALT—Alanine Aminotransferase; AST—Aspartate Aminotransferase; ALP—Alkaline Phosphatase; GGT—Gamma Glutamyl Transpeptidase; LDH—Lactate Dehydrogenase; PT—Prothrombin Time; APTT—Activated Partial Thromboplastin clotting Time; FIB-4—Fibrosis 4 score; NFS—nonalcoholic fatty liver disease fibrosis score; APRI—AST to Platelet Ratio Index.

**Table 3:** Comparison of biological parameters six months after hospitalization between cases and controls.

Variables (Median IQR)	Normal Range	Long COVID <65 Years (*n* = 117)	Long COVID ≥65 Years (*n* = 71)	No Long COVID (*n* = 50)	*p*-Value
Fasting glucose (mmol/L)	60–125	119.2 (109.3–129.8)	126.7 (116.9–137.2)	97.8 (88.1–107.9)	<0.001
ALT (U/L)	7–35	49.7 (40.2–60.1)	58.2 (48.9–68.4)	28.5 (19.1–38.6)	<0.001
AST (U/L)	10–40	51.8 (42.5–62.4)	62.3 (53.1–72.8)	33.9 (24.7–44.6)	<0.001
ALP (U/L)	40–130	145.9 (135.7–157.6)	164.3 (153.8–176.5)	110.2 (100.1–121.8)	<0.001
Serum albumin (g/dL)	3.4–5.4	3.7 (3.5–3.9)	3.3 (3.1–3.6)	4.2 (4.0–4.5)	0.038
Total proteins (g/dL)	6.0–8.3	7.2 (6.9–7.6)	6.7 (6.3–7.1)	7.8 (7.5–8.2)	0.022
Total bilirubin (g/dL)	0.3–1.2	1.3 (1.1–1.6)	1.6 (1.3–1.9)	0.8 (0.6–1.0)	0.064
GGT (U/L)	0–30	36.4 (31.1–42.8)	41.9 (36.3–48.2)	24.6 (19.4–30.5)	<0.001
LDH (U/L)	140–280	292.7 (276.4–310.3)	315.8 (299.2–333.9)	210.4 (194.1–228.2)	<0.001
PT (seconds)	11.0–13.5	14.3 (13.7–15.0)	15.4 (14.8–16.1)	12.6 (12.0–13.3)	<0.001
APTT (seconds)	30–40	39.5 (37.2–41.9)	42.8 (40.4–45.3)	33.1 (30.8–35.6)	<0.001
FIB-4	1.45–3.25	1.9 (1.6–2.3)	2.7 (1.9–3.6)	1.4 (1.1–1.8)	0.010
NFS	≤1.5	−0.9 (−1.3–−0.6)	−0.5 (−0.9–−0.2)	−1.7 (−2.0–−1.5)	0.073
APRI	0.5–1.5	1.1 (0.9–1.4)	1.5 (1.1–1.9)	0.7 (0.5–0.9)	0.055

IQR—Interquartile Range; ALT—Alanine Aminotransferase; AST—Aspartate Aminotransferase; ALP—Alkaline Phosphatase; GGT—Gamma Glutamyl Transpeptidase; LDH—Lactate Dehydrogenase; PT—Prothrombin Time; APTT—Activated Partial Thromboplastin clotting Time; FIB-4—Fibrosis 4 score; NFS—nonalcoholic fatty liver disease fibrosis score; APRI—AST to Platelet Ratio Index.

**Table 4:** Intragroup comparison between Long COVID cases at admission and six months after discharge.

Variables (Median IQR)	Normal Range	Initial Measurement	Second Measurement	*p*-Value
Long COVID <65 years (*n* = 117)				
Fasting glucose (mmol/L)	60–125	132.4 (121.3–143.7)	119.2 (109.3–129.8)	<0.001
ALT (U/L)	7–35	61.7 (51.2–72.9)	49.7 (40.2–60.1)	<0.001
AST (U/L)	10–40	71.2 (61.4–81.9)	51.8 (42.5–62.4)	<0.001
ALP (U/L)	40–130	163.5 (152.3–175.8)	145.9 (135.7–157.6)	0.004
Serum albumin (g/dL)	3.4–5.4	2.6 (2.4–2.9)	3.7 (3.5–3.9)	<0.001
Total proteins (g/dL)	6.0–8.3	6.7 (6.3–7.2)	7.2 (6.9–7.6)	0.058
Total bilirubin (g/dL)	0.3–1.2	1.8 (1.5–2.2)	1.3 (1.1–1.6)	0.106
GGT (U/L)	0–30	45.3 (39.8–52.1)	36.4 (31.1–42.8)	0.001
LDH (U/L)	140–280	312.4 (295.1–331.7)	292.7 (276.4–310.3)	0.042
PT (seconds)	11.0–13.5	15.2 (14.6–15.9)	14.3 (13.7–15.0)	0.094
APTT (seconds)	30–40	43.8 (41.5–46.4)	39.5 (37.2–41.9)	0.010
FIB-4	1.45–3.25	3.3 (2.0–4.7)	1.9 (1.6–2.3)	<0.001
NFS	<-1.5	−0.4 (−0.7–−0.1)	−0.9 (−1.3–−0.6)	<0.001
APRI	0.5–1.5	1.8 (1.0–2.5)	1.1 (0.9–1.4)	0.003
Long COVID ≥65 years (*n* = 71)				
Fasting glucose (mmol/L)	60–125	147.8 (136.5–158.2)	126.7 (116.9–137.2)	<0.001
ALT (U/L)	7–35	82.3 (71.8–93.1)	58.2 (48.9–68.4)	<0.001
AST (U/L)	10–40	92.5 (82.7–103.6)	62.3 (53.1–72.8)	<0.001
ALP (U/L)	40–130	194.7 (182.4–207.9)	164.3 (153.8–176.5)	<0.001
Serum albumin (g/dL)	3.4–5.4	2.1 (1.9–2.4)	3.3 (3.1–3.6)	<0.001
Total proteins (g/dL)	6.0–8.3	5.9 (5.4–6.5)	6.7 (6.3–7.1)	0.040
Total bilirubin (g/dL)	0.3–1.2	2.4 (2.0–2.9)	1.6 (1.3–1.9)	0.033
GGT (U/L)	0–30	58.6 (51.9–66.4)	41.9 (36.3–48.2)	<0.001
LDH (U/L)	140–280	364.5 (345.9–384.8)	315.8 (299.2–333.9)	0.002
PT (seconds)	11.0–13.5	16.7 (16.0–17.5)	15.4 (14.8–16.1)	0.065
APTT (seconds)	30–40	49.3 (46.9–52.1)	42.8 (40.4–45.3)	0.001
FIB-4	1.45–3.25	3.9 (2.5–4.4)	2.7 (1.9 −3.6)	<0.001
NFS	≤1.5	0.2 (−0.1–0.6)	−0.5 (−0.9–−0.2)	0.010
APRI	0.5–1.5	2.6 (1.3–3.9)	1.5 (1.1–1.9)	<0.001

IQR—Interquartile Range; ALT—Alanine Aminotransferase; AST—Aspartate Aminotransferase; ALP—Alkaline Phosphatase; GGT—Gamma Glutamyl Transpeptidase; LDH—Lactate Dehydrogenase; PT—Prothrombin Time; APTT—Activated Partial Thromboplastin clotting Time; FIB-4—Fibrosis 4 score; NFS—nonalcoholic fatty liver disease fibrosis score; APRI—AST to Platelet Ratio Index.

**Table 5:** Comparison of differences measured in laboratory values from admission to six months post discharge.

Variables (Median-IQR)	Long COVID <65 Years (*n* = 117)	Long COVID ≥65 Years (*n* = 71)	No Long COVID (*n* = 50)	*p*-Value
ΔFasting glucose (mmol/L)	13.2 (11.0–15.9)	21.1 (18.6–24.3)	14.7 (12.4–17.5)	<0.001
ΔALT (U/L)	12.0 (9.0–15.6)	24.1 (19.7–29.4)	18.9 (15.0–23.5)	<0.001
ΔAST (U/L)	19.4 (16.9–22.4)	30.2 (26.6–34.1)	18.4 (15.2–21.9)	<0.001
ΔALP (U/L)	17.6 (15.6–20.2)	30.4 (27.6–33.9)	33.0 (29.5–37.1)	<0.001
ΔSerum albumin (g/dL)	−1.1 (−1.3–−0.9)	−1.2 (−1.4–−1.0)	−1.0 (−1.2–−0.8)	0.092
ΔTotal proteins (g/dL)	−0.5 (−0.7–−0.3)	−0.8 (−1.0–−0.6)	−0.5 (−0.7–−0.3)	0.059
ΔTotal bilirubin (g/dL)	0.5 (0.3–0.7)	0.8 (0.6–1.0)	0.3 (0.1–0.5)	0.034
ΔGGT (U/L)	8.9 (6.7–11.3)	16.7 (13.9–19.8)	6.9 (4.8–9.5)	<0.001
ΔLDH (U/L)	19.7 (16.2–23.5)	48.7 (43.1–55.0)	54.9 (49.5–61.2)	<0.001
ΔPT (seconds)	0.9 (0.7–1.2)	1.3 (1.0–1.6)	0.8 (0.5–1.0)	0.009
ΔAPTT (seconds)	4.3 (3.6–5.1)	6.5 (5.7–7.4)	3.6 (3.0–4.3)	<0.001
ΔFIB-4	1.4 (1.1–1.8)	1.2 (0.9–1.6)	0.3 (0.2–0.5)	0.010
ΔNFS	0.5 (0.3–0.7)	0.7 (0.5–0.9)	−0.1 (−0.3–0.1)	0.041
ΔAPRI	0.7 (0.5–0.9)	1.1 (0.8–1.4)	0.1 (−0.1–0.3)	<0.001

Δ = admission values minus six months post-discharge values; IQR—Interquartile Range; ALT—Alanine Aminotransferase; AST—Aspartate Aminotransferase; ALP—Alkaline Phosphatase; GGT—Gamma Glutamyl Transpeptidase; LDH—Lactate Dehydrogenase; PT—Prothrombin Time; APTT—Activated Partial Thromboplastin clotting Time; FIB-4—Fibrosis 4 score; NFS—nonalcoholic fatty liver disease fibrosis score; APRI—AST to Platelet Ratio Index.

**Table 6:** Regression analysis of factors predicting liver injury severity post SARS-CoV-2 infection.

Independent Variables	Coefficient (β)	Std. Error	*p*-Value	95% CI
Demographic Factors				
Age (years)	0.048	0.014	<0.001	[0.021, 0.075]
Gender (1 = Male, 0 = Female)	−0.107	0.192	0.558	[−0.484, 0.270]
BMI (kg/m²)	0.023	0.015	0.148	[−0.007, 0.053]
Clinical Factors				
Smoking (1 = Yes, 0 = No)	0.293	0.244	0.223	[−0.186, 0.772]
Cardiovascular disease (1 = Yes, 0 = No)	0.429	0.217	0.046	[0.004, 0.854]
Chronic kidney disease (1 = Yes, 0 = No)	0.574	0.307	0.069	[−0.028, 1.176]
CCI score (≥2)	0.149	0.074	0.041	[0.004, 0.294]
Biological Parameters Change (Δ)				
AST (U/L)	0.036	0.009	<0.001	[0.018, 0.054]
ALP (U/L)	0.049	0.012	<0.001	[0.036, 0.075]
Total bilirubin (g/dL)	1.198	0.400	0.003	[0.413, 1.983]
GGT (U/L)	0.017	0.007	0.028	[0.003, 0.031]
LDH (U/L)	0.005	0.002	0.012	[0.001, 0.009]
Serum albumin (g/dL)	−1.503	0.513	0.005	[−2.509, −0.497]
PT (seconds)	0.210	0.101	0.037	[0.012, 0.408]
APTT (seconds)	−0.324	0.153	0.034	[−0.624, −0.024]
FIB-4	0.202	0.104	0.053	[0.001, 0.403]
NFS	−0.315	0.150	0.032	[−0.609, −0.021]
APRI	0.138	0.065	0.038	[0.010, 0.266]
Treatment Variables				
Oxygen supplementation (1 = Yes, 0 = No)	−0.140	0.203	0.489	[−0.538, 0.258]
ICU admission (1 = Yes, 0 = No)	0.483	0.235	0.042	[0.022, 0.944]

CI—Confidence Interval; BMI—Body Mass Index; ICU—Intensive Care Unit; CCI—Charlson Comorbidity Index; AST—Aspartate Aminotransferase; ALP—Alkaline Phosphatase; GGT—Gamma Glutamyl Transpeptidase; LDH—Lactate Dehydrogenase; PT—Prothrombin Time; APTT—Activated Partial Thromboplastin clotting Time; FIB-4—Fibrosis 4 score; NFS—nonalcoholic fatty liver disease fibrosis score; APRI—AST to Platelet Ratio Index.

## Data Availability

The data presented in this study are available on request from the corresponding author.
